# Mitochondrial hyperpolarization in iPSC-derived neurons from patients of FTDP-17 with 10+16 *MAPT* mutation leads to oxidative stress and neurodegeneration

**DOI:** 10.1016/j.redox.2017.03.008

**Published:** 2017-03-10

**Authors:** Noemí Esteras, Jonathan D. Rohrer, John Hardy, Selina Wray, Andrey Y. Abramov

**Affiliations:** a Department of Molecular Neuroscience, UCL Institute of Neurology, Queen Square, WC1N 3BG London, UK; b Dementia Research Centre, UCL Institute of Neurology, London, UK

**Keywords:** 2-NBDG, 2-(N-(7-Nitrobenz-2-oxa-1,3-diazol-4-yl)Amino)-2-Deoxyglucose, AD, Alzheimer's disease, CBD, Corticobasal Degeneration, CSF, Cerebrospinal Fluid, DHE, Dihydroethidium, ETC, Electron Transport Chain, FCCP, Carbonyl cyanide-4-(trifluoromethoxy)phenylhydrazone, FTD, Frontotemporal dementia, FTDP-17, Frontotemporal Dementia with Parkinsonism linked to chromosome 17, hESC, Human Embryonic Stem Cells, IAA, Iodoacetic Acid, iPSC, Induced Pluripotent Stem Cells, NaPyr, Sodium Pyruvate, OXPHOS, Oxidative Phosphorylation, PDH, Pyruvate Dehydrogenase, PDK, Pyruvate Dehydrogenase Kinase, PSP, Progressive Nuclear Palsy, ROS, Reactive Oxygen Species, TCA cycle, tricarboxylic acid cycle, TMRM, tetramethylrhodamine methyl ester, VDAC, Voltage Dependent Anion Channel, ΔΨ_m_, Mitochondrial Membrane Potential, Mitochondria, iPSC‐derived neurons, Tau, Glycolysis, Hyperpolarization, Oxidative stress

## Abstract

Tau protein inclusions are a frequent hallmark of a variety of neurodegenerative disorders. The 10+16 intronic mutation in *MAPT* gene, encoding tau, causes frontotemporal dementia and parkinsonism linked to chromosome 17 (FTDP-17), by altering the splicing of the gene and inducing an increase in the production of 4R tau isoforms, which are more prone to aggregation. However, the molecular mechanisms linking increased 4R tau to neurodegeneration are not well understood.

Here, we have used iPSC-derived neurons from patients of FTDP-17 carrying the 10+16 mutation to study the molecular mechanisms underlying neurodegeneration.

We show that mitochondrial function is altered in the neurons of the patients. We found that FTDP-17 neurons present an increased mitochondrial membrane potential, which is partially maintained by the F1Fo ATPase working in reverse mode. The 10+16 *MAPT* mutation is also associated with lower mitochondrial NADH levels, partially supressed complex I-driven respiration, and lower ATP production by oxidative phosphorylation, with cells relying on glycolysis to maintain ATP levels. Increased mitochondrial membrane potential in FTDP-17 neurons leads to overproduction of the ROS in mitochondria, which in turn causes oxidative stress and cell death. Mitochondrial ROS overproduction in these cells is a major trigger for neuronal cell death and can be prevented by mitochondrial antioxidants

## Highlights

• iPSC-derived neurons with 10+16 *MAPT* mutation show altered bioenergetics.
• Respiration is inhibited in patients’ neurons, but ATP is compensated by glycolysis.
• Bioenergetic alterations can be rescued with substrates for respiration.
• Mitochondrial membrane potential is higher, leading to oxidative stress and cell death.

## Graphical abstract

**Figure:**
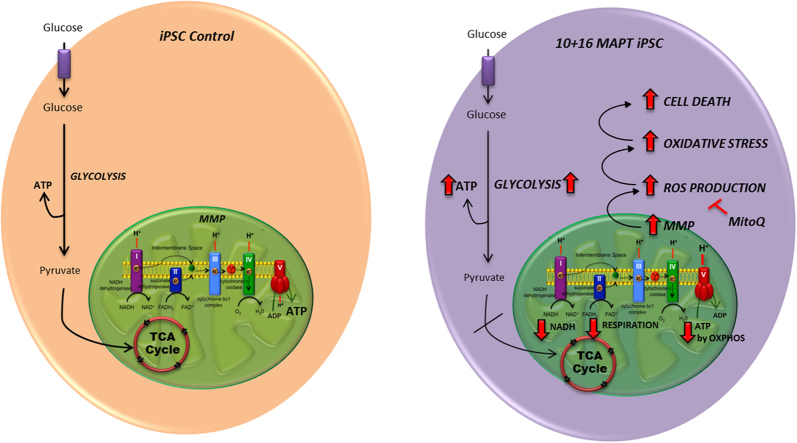

## 1 Introduction

Tau protein is implicated in the pathogenesis of several clinically diverse neurodegenerative disorders collectively termed the tauopathies. Although clinically heterogeneous, ranging from dementias such as Alzheimer's disease (AD) or frontotemporal dementia (FTD) to movement disorders such as progressive supranuclear palsy (PSP) or corticobasal degeneration (CBD), they are all characterized by the deposition of abnormal tau protein in different regions of the brain [40]. Tau is mostly expressed in the nervous system, especially in neurons, and is implicated in microtubule assembly and stabilization [44], being essential for morphogenesis, axonal extension or axonal transport [4].

There are six isoforms of the protein expressed in the adult human brain, generated by alternative splicing of exons 2, 3 and 10 of *MAPT*, the gene that encodes tau [15]. The inclusion of exon 10 leads to tau isoforms containing 4 microtubule-binding repeats (4R), while its exclusion generates the 3R isoforms, containing 3 microtubule-binding repeats. In a normal brain there is a similar proportion of 3R and 4R isoforms [13], [23]. However, certain *MAPT* mutations can cause increased exon 10 inclusion, resulting in an overproduction of 4R isoforms and an imbalance of the 3R/4R ratio, which is enough to cause neurodegeneration and dementia [28]. This is the case of the intronic 10+16 *MAPT* mutation, that causes frontotemporal dementia with parkinsonism linked to chromosome 17 (FTDP-17). An imbalance in the 3R/4R ratio is also observed in the sporadic tauopathies PSP and CBD.

The potential of induced pluripotent stem cells (iPSC) technology to develop human neuronal models directly from patient's cells has become a very useful tool to understand the pathogenic mechanisms of neurodegenerative disorders linked to genetic mutations [32]. We have recently shown that iPSC-derived cortical neurons from patients with the 10+16 mutation in *MAPT* express both 3R and 4R tau, in contrast to control neurons which express mainly 3R tau at early stages of development [41]. At extended time-points, iPSC-derived neurons recapitulate the tau expression and splicing patterns observed in human brain development. Thus, this model represents a promising approach for the study of the molecular mechanisms of tau pathology occurring in FTD.

One of the several factors that can lead to neuronal loss in neurodegenerative disorders is the dysfunction of the mitochondria. In the present study we were focused on the analysis of the mitochondrial function and bioenergetics in iPSC-derived neurons from patients of FTDP-17 carrying the *MAPT* 10+16 mutation. We show that the 10+16 *MAPT* mutation induces an impairment of the mitochondrial function: neurons from the patients present alterations in the bioenergetics, with a reduced NADH mitochondrial pool resulting in decreased respiration, and a reduction of the ATP produced by oxidative phosphorylation which is compensated by an increased production of ATP by glycolysis. 10+16 *MAPT* mutation is also associated with a higher mitochondrial membrane potential that leads to an increase in ROS production, oxidative stress and ultimately cell death.

## 2 Materials and methods

### 2.1 Cell lines

Experiments were performed with 4 different cell lines. The hESC control line Shef6 was obtained from UK Stem Cell Bank. The two patient iPSC lines were generated by retroviral-transduction reprogramming of fibroblasts obtained from the National Hospital for Neurology and Neurosurgery and the control iPSC line, also generated using retroviral transduction, was obtained from the laboratory of Dr Tilo Kunath. All the information regarding the reprogramming of fibroblasts into iPSC and characterization of the lines can be found in [41].

Differentiation of the iPSC into cortical neurons was done using dual SMAD inhibition followed by *in vitro* neurogenesis as described in [37], [41].

30–40 days after induction, cells were plated in poly-ornithine/laminin coated μ-Slide 8 well Ibidi chambers (Martinsried, Germany). Cells were then maintained in neural maintenance media described in [37], with media changes twice a week. All the experiments were performed in neurons older than 80 days (after induction).

### 2.2 Live imaging

All experiments were done in commercial HBSS 1X (with calcium and magnesium) (ThermoFisher) supplemented with 10 mM HEPES and adjusted to pH 7.4.

#### 2.2.1 Measurement of mitochondrial membrane potential

Cells were incubated at room temperature for 40 min with 25 nM tetramethylrhodamine methyl ester (TMRM), and when necessary for mitochondrial mass measurements with Calcein-AM (1 μM, Molecular Probes, ThermoFisher) and 0.005% Pluronic. Images were acquired using a Zeiss 710 VIS CLMS confocal microscope equipped with a META detection system and an x40 oil immersion objective (Zeiss, Oberkochen, Germany). The 560 nm laser line was used to excite the TMRM and the 488 nm to excite calcein. Emitted fluorescence was measured above 580 nm for TMRM and between 500 and 540 nm for calcein, keeping the power laser at minimum to avoid phototoxicity. Z-stacks were acquired for the calculations of the mitochondrial membrane potential and mass. Images were analysed using Volocity 3D Image Analysis Software (PerkinElmer, Waltham, MA, USA). Data was obtained from n=9–38 Z-Stacks in 6–12 independent experiments done with different inductions, and was normalized to control cells in all of them. For ΔΨm maintenance experiments, a single focal plane was selected and TMRM intensity was measured during the appropriate amount of time. TMRM was used in the redistribution mode, so a reduction in TMRM signal represents mitochondrial depolarization. Zeiss software was used for the analysis of the experiments. Basal TMRM levels were taken as 100% and remaining TMRM fluorescence in the mitochondria after complete depolarization caused by FCCP was taken as 0%. Data was obtained from n=26–40 cells analysed in 3–5 independent experiments done with different inductions.

#### 2.2.2 Measurement of NADH redox state

NADH autofluorescence was measured using an epifluorescence-inverted microscope equipped with a x40 oil objective. A Xenon arc lamp passed through a monochromator was used to provide excitation light at 360 nm. Emitted light was reflected through a 455 nm long-pass filter to a cooled CCD camera. Images were acquired and analysed using Andor Software. After recording basal autofluorescence, 1 μM FCCP was added to completely depolarize the mitochondria and oxidize the mitochondrial pool of NADH to NAD^+^, which is no longer fluorescent. This point was taken as 0%. 1 mM NaCN was then added to inhibit respiration and allow the regeneration of the mitochondrial pool of NADH (100%). NADH pool was calculated as the difference between maximum and minimum values of autofluorescence in the mitochondria. NADH redox index was calculated as the % represented by the basal levels when extrapolating its value in the 0–100% range generated by FCCP and NaCN respectively. A total number of n=100–200 cells were analysed in 4–7 independent experiments.

#### 2.2.3 Measurement of glucose uptake

The fluorescent glucose analogue 2-NBDG (Molecular Probes, Thermofisher) was used for glucose uptake experiments. Cells were incubated in HBSS containing no glucose in the presence of 2 mM 2-NBDG for 25 min. Cells were then washed and 4–5 z-stacks per well were acquired using a Zeiss 710 VIS CLMS confocal microscope equipped with a META detection system and an x40 oil immersion objective (Zeiss, Oberkochen, Germany). The 488 nm laser was used to excite 2-NBDG and emitted fluorescence was measured above 495 nm. Green fluorescence inside the cells was quantified using Zeiss software. At least 90 cells per line were analysed in a minimum of 5 independent measurements.

#### 2.2.4 Measurement of ATP

For the monitoring of ATP levels, cells were transfected with the mitochondrial-targeted ATP indicator AT1.03, designed by [20]. This genetically encoded FRET indicator allows the visualization of the dynamics of ATP in real time. Briefly, this probe contains a variant of the cyan fluorescent protein (CFP) and a variant of yellow fluorescence protein (YFP). When ATP is not bound to the probe, both fluorescent proteins are separated and the FRET efficiency is low, so fluorescence emitted from the CFP is the mainly detected. In contrast, when ATP binds to the probe, the two fluorescence proteins come close to each other, increasing FRET efficiency, and as a result, YFP signal. Thus, the YFP/CFP emission ratio is used to evaluate ratiometrically the levels of ATP. Measurement of ATP levels with mitoAT1.03 probe was performed on the confocal microscope Zeiss 710 LSM with an integrated META-detection system using a 40x oil-immersion objective. CFP was excited with 405 nm laser, and its emission was detected between 460–510 nm. Emission from YFP was detected between 540–600 nm.

Cells were transfected using the Effectene transfection reagent from Qiagen (Hilden, Germany), following the manufacturer's indications. AT1.03 cDNA was kindly provided by Prof Imamura from the Habuki Center & Graduate School of Biostudies, (Kyoto University, Japan). Data was obtained from n=13–37 cells in 4–7 independent experiments, and was normalized to control cells in all of them.

#### 2.2.5 ROS production and lipid peroxidation

Cytosolic ROS production was monitored in single cells using the superoxide indicator dihydroethidium (DHE, 2 μM, Molecular Probes, ThermoFisher), which shows blue fluorescence in the cytosol until oxidized, when it intercalates within the DNA, staining the nucleus fluorescent red. Live-imaging experiments were performed in an epifluorescence inverted microscope using a Xenon arc lamp passed through a monochromator to provide excitation light at 530 nm. Emitted light was reflected through a 605 nm long-pass filter to a cooled CCD camera. Cells were loaded with DHE and basal rate of ROS production, measured as the rate of increase in red DHE fluorescence, was immediately recorded for several minutes. Images were captured and analysed using IQ2 software from Andor. Around n=100–200 cells were analysed in at least four independent experiments.

Mitochondrial ROS production was analysed using the mitochondrial-targeted dye MitoTracker® Red CM-H2XRos (Molecular Probes, Thermofisher). Cells were loaded for 20 min with the dye, and measurements were done on the confocal microscope Zeiss 710 LSM with an integrated META-detection system using a 40x oil-immersion objective. The dye was excited with 561 nm laser, and emission was detected above 580 nm and recorded for several minutes. The rate of increase in red fluorescence for each whole field was analysed using Volocity 3D Image Analysis Software (PerkinElmer, Waltham, MA, USA). N=4–11 fields containing at least 100 cells each were analysed in 4–6 independent experiments.

Lipid peroxidation was measured in single cells with Bodipy 581/591 C11 (2 μM, Molecular Probes, ThermoFisher) on the confocal microscope Zeiss 710 LSM with an integrated META-detection system using a 40x oil-immersion objective. Bodipy 581/591 C11 is a fluorescent fatty acid analogue that incorporates into cellular membranes and shows fluorescence in the red range of the spectrum (~590 nm). Upon oxidation, the fluorescent properties shift from red to green (~520 nm), allowing the ratiometric measurement of lipid peroxidation. Bodipy was excited using the 488 nm and 561 nm laser lines and fluorescence detected in 500–550 nm and 575–680 nm. Images were acquired and analysed using Zeiss software. The rate of the increase of the Bodipy ratio (green/red) was calculated in n=50–200 cells in at least 3 different experiments.

#### 2.2.6 Cell death

Cells were loaded with 20 μM propidium iodide and 10 μM Hoechst 33342. While Hoechst is a blue fluorescent dye that stains chromatin DNA, propidium iodide is only permeable to dead cells and shows red fluorescence, so it is possible to calculate the percentage of dead cells (showing red fluorescence) *vs.* total number of cells (showing blue fluorescence). Images were acquired in the Zeiss 710 LSM confocal microscope with an integrated META-detection system using a 20x objective and analysed using ImageJ software. A total number of 500–2000 cells were counted in n=18–28 different fields. Experiments were repeated at least 3 times with different inductions.

### 2.3 Statistics

All histograms represent the average±SEM. Statistical analysis (One-way ANOVA followed by Tukey post-hoc correction) was performed in OriginPro 2016 software. Differences were considered to be significant if *p*<0.05.

## 3 Results

### 3.1 10+16 *MAPT* mutation is associated with higher mitochondrial membrane potential partially maintained by complex V

To study if the *MAPT* 10+16 mutation affects mitochondrial function, we used as a model iPSC-derived neurons from two FTDP-17 patients carrying the specific intronic mutation together with a control iPSC line and from a human embryonic stem cell line (hESC) [41].

**Fig. 1:**
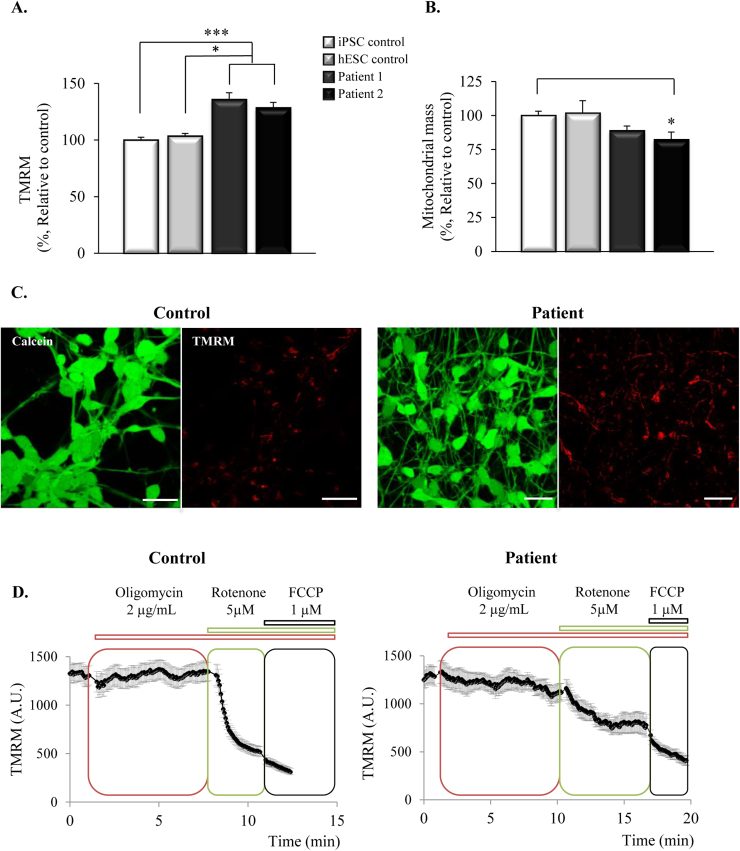
**Mitochondrial membrane potential is higher in patients with the*****MAPT*****10+16 mutation and is partially maintained by complex V working in reverse mode**. **1A**. Mitochondrial membrane potential (ΔΨm) was estimated using tetramethylrhodamine methyl ester (TMRM) indicator. Neurons from the patients with 10+16 *MAPT* mutation showed a significant increase in ΔΨ_m_ compared to controls (*p<0.05, ***p<0.001). **1B**. Mitochondrial mass was lower in the neurons from the patient (*p<0.05) as estimated by the percentage of the cell occupied by mitochondria (stained with TMRM) in relation to the total volume of the cell (stained with calcein). **1C**. Representative images from live imaging experiments showing the TMRM (red) and calcein (green) staining. Scale bar: 20 µm. **1D**. Time-course experiments of TMRM signal showing the maintenance of the ΔΨm in the presence of inhibitors of different mitochondrial complexes. Oligomycin (inhibitor of complex V) didn’t affect the ΔΨm in the control cells but induced a partial depolarization in patient cells. Inhibition of complex I with rotenone induced a strong depolarization in control cells that was much smaller in patient's cells, and FCCP led to the complete depolarization in both cases, showing that contribution of complex II to the maintenance of ΔΨm was higher in patient cells than in controls.

We first measured mitochondrial membrane potential (ΔΨ_m_) as it represents a good indicator of the health of the cells and alterations in ΔΨ_m_ are frequently found in neurodegenerative disorders. We loaded the cells with tetramethylrhodamine methyl ester (TMRM) and used live imaging to estimate the ΔΨ_m_. Surprisingly, we found that the iPSC-derived neurons from the patients of FTDP-17 show an increase in ΔΨ_m_ compared to both iPSC and hESC-derived neurons from healthy controls, as suggested by the significantly higher TMRM signal (Fig. 1A). Compared to the control lines (100±2.5% (iPSC control, n=47) and 103.5±2.3% (hESC control, n=9)) the neurons from the patients with the mutation in *MAPT* showed an increase in TMRM intensity to 135.8±6% (patient 1, n=38) and 128.6±4.6% (patient 2, n=19). The mitochondrial mass, calculated as the percentage of the volume occupied by mitochondria (stained with TMRM) in the total volume of the cell (stained with calcein), appeared to be lower in the patients (Fig. 1B) (patient 1, 88.9±3.4%, n=14; patient 2, 82.2±5.7%, n=11) compared to the controls (100±3.2%, n=18 and 101.8±9.1%, n=3; Fig. 1B). Representative images of calcein and TMRM staining can be found in Fig. 1C.

We next examined the mechanism by which ΔΨ_m_ was maintained in these cells. In healthy mitochondria, ΔΨ_m_ is maintained by the pumping of protons from the matrix to the intermembrane space through the complexes of the electron transport chain. The protons then cross back to the matrix through the ATP synthase (complex V), and the energy derived from the proton-motive force is used to generate ATP. However, in case of pathology, complex V can work in reverse mode, hydrolysing ATP and pumping protons to the intermembrane space. To test the contribution of each of the complexes to the maintenance of the ΔΨ_m,_ we first treated the neurons with the complex V inhibitor, oligomycin (2 μg/mL). As shown in Fig. 1D, control iPSC-derived neurons showed no response to oligomycin (2.95±3.76% decrease of the TMRM signal, n=40), while patients showed a significant decrease in the TMRM signal, suggesting that in these cells, complex V is working in reverse to maintain the ΔΨ_m_ (patient 1, 28.4±4.8%, n=26; patient 2, 19.9±3.6%, n=29). The subsequent inhibition of complex I by rotenone (5 μM) caused a deep decrease in the TMRM signal in healthy controls (78.2±4.1), that was much smaller in the patients (patient 1, 41.9±3.4%; patient 2, 43.5±2.9%). Treatment of the cells with the uncoupler FCCP (1 μM) completely depolarised the mitochondria, causing a further decrease in the ΔΨ_m_ which indicates the contribution of complex II. In the cells from the patients, the response to FCCP appeared to be higher than in control cells, suggesting a possible compensating mechanism of complex II for the decreased contribution of complex I (control, 19.9±3.6%; patient 1, 43.5±2.9%; patient 2, 36.7±3.7%).

These results indicate that the electron transport chain activity is not sufficient to maintain the ΔΨ_m_ in the cells of the patients with the mutation in *MAPT*. Instead, complex V working in reverse partially maintains the ΔΨm, with reduced complex I and increased complex II contributions.

### 3.2 The *MAPT* 10+16 mutation is associated with a more reduced NADH redox state and lower mitochondrial NADH pool

**Fig. 2:**
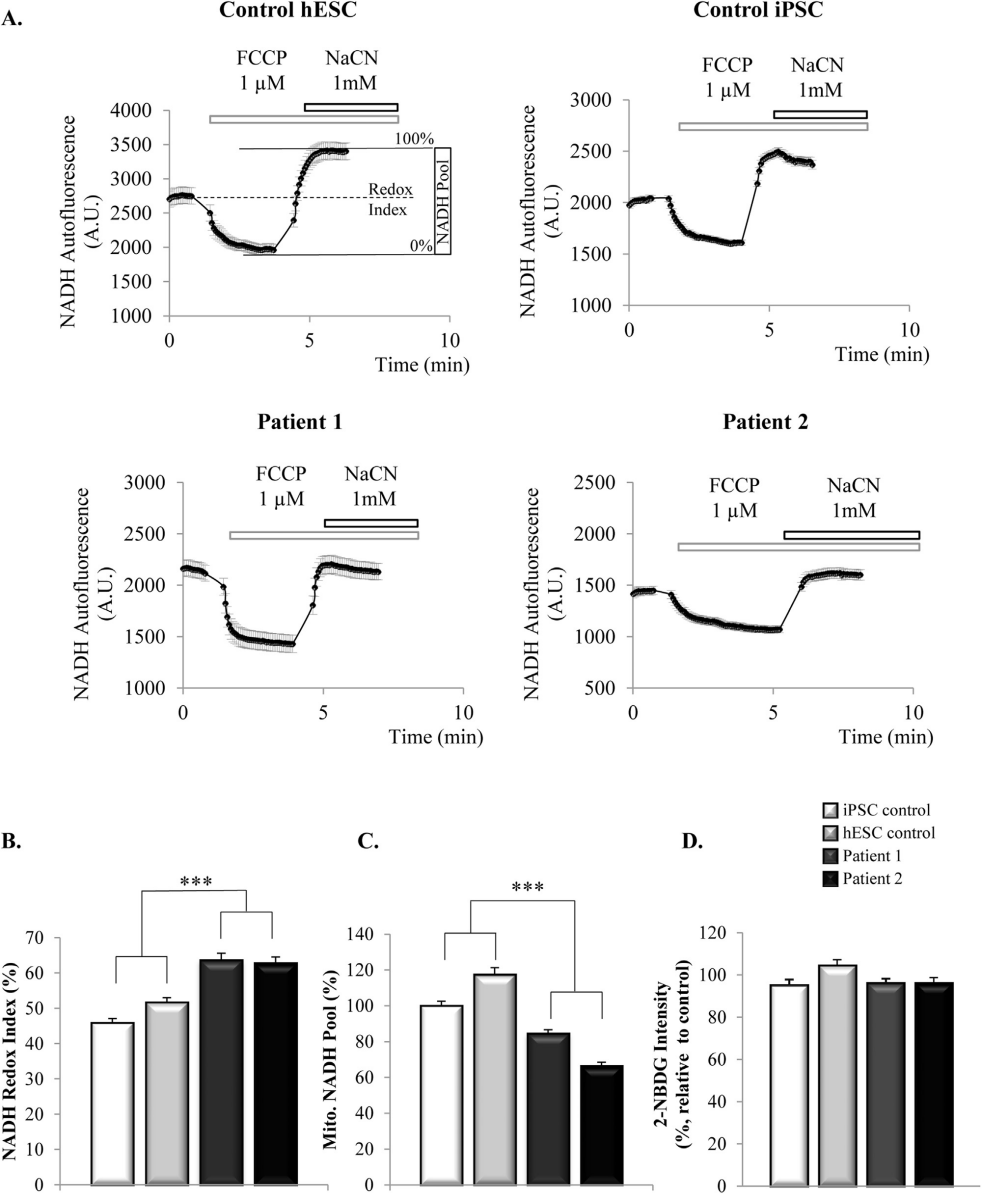
**Mitochondrial NADH pool is lower and complex I-driven respiration is inhibited in iPSC-derived neurons from patients with the*****MAPT*****10+16 mutation**. **2A**. Representative traces of NADH status experiments. Basal autofluorescence of NADH was measured prior to the addition of FCCP to complete depolarize the mitochondria, stimulating respiration and causing the complete oxidation of NADH to NAD^+^, which is no longer fluorescent. This point was considered as 0%. NaCN was then added to inhibit respiration and allow the complete regeneration of mitochondrial NADH pool (maximally reduced), taken as 100%. Basal NADH redox state (%) was extrapolated from the 0–100% range and mitochondrial NADH pool was calculated as the difference between min and max autofluorescence. **2B**. NADH Redox Index is higher (*i.e.* more reduced) in patients carrying the 10+16 *MAPT* mutation (***p<0.001), suggesting the inhibition of complex I-driven respiration. **2C**. Mitochondrial NADH pool is lower in neurons from the patients (***p<0.001). **2D**. Quantification of glucose uptake by the cells using the fluorescent glucose analogue 2-NBDG as described in Methods.

We next examined the respiratory function by analysing the redox state of NADH, as an indicator of the activity of respiratory complex I, representative traces are shown in Fig. 2A. NADH redox state was calculated as the percentage of the basal mitochondrial NADH autofluorescence, in a scale were 0% represents the maximally oxidized NADH (obtained after depolarising the mitochondria with FCCP 1 µM) and 100% represents the maximally reduced NADH (obtained after inhibiting respiration with NaCN 1 mM, and therefore allowing the regeneration of the mitochondrial pool of NADH), as described in [5]. Cells from patients show a significantly higher (more reduced) redox index than control cells (Fig. 2B), suggesting that complex I-driven respiration is inhibited in mutant cells compared to the controls (iPSC control, 45.9±1.1%, n=212 cells; hESC control, 51.7±1.3%, n=108 cells; patient 1, 63.7±1.9%, n=127 cells; patient 2, 62.9±1.6%, n=103 cells). One of the reasons that may explain this situation could be a lower availability of substrates for respiration coming from the TCA cycle. Indeed, as presented in Fig. 2C, cells from patients show a lower total mitochondrial NADH pool than control cells (iPSC control, 100±2.5%; hESC control, 117±3.7%; patient 1, 84.5±2.1; patient 2, 66.5±2.2), which suggests that inhibition of respiration in patient cells may be due to a diminished availability of NADH for complex I.

One of the reasons for the lower availability of substrates for respiration could be an alteration in the glucose uptake in these cells that may affect the downstream metabolism. To investigate it, we studied the uptake of the fluorescent glucose analogue 2-NBDG (2 mM) by the cells in HBSS media containing no glucose. As shown in Fig. 2D, the uptake of 2-NBDG was similar in all the cell lines (control iPSC 95.2±2.7%, n=115; control hESC 104.5±2.7%, n=118; patient 1 96.4±2%, n=101; patient 2 96.3±2.3%, n=90), excluding the possibility of an altered glucose uptake in these cells as the reason for the bioenergetics alterations found.

### 3.3 ATP levels are similar in control and patient cells, but glycolysis is the main source of ATP in the cells with the *MAPT* 10+16 mutation

**Fig. 3:**
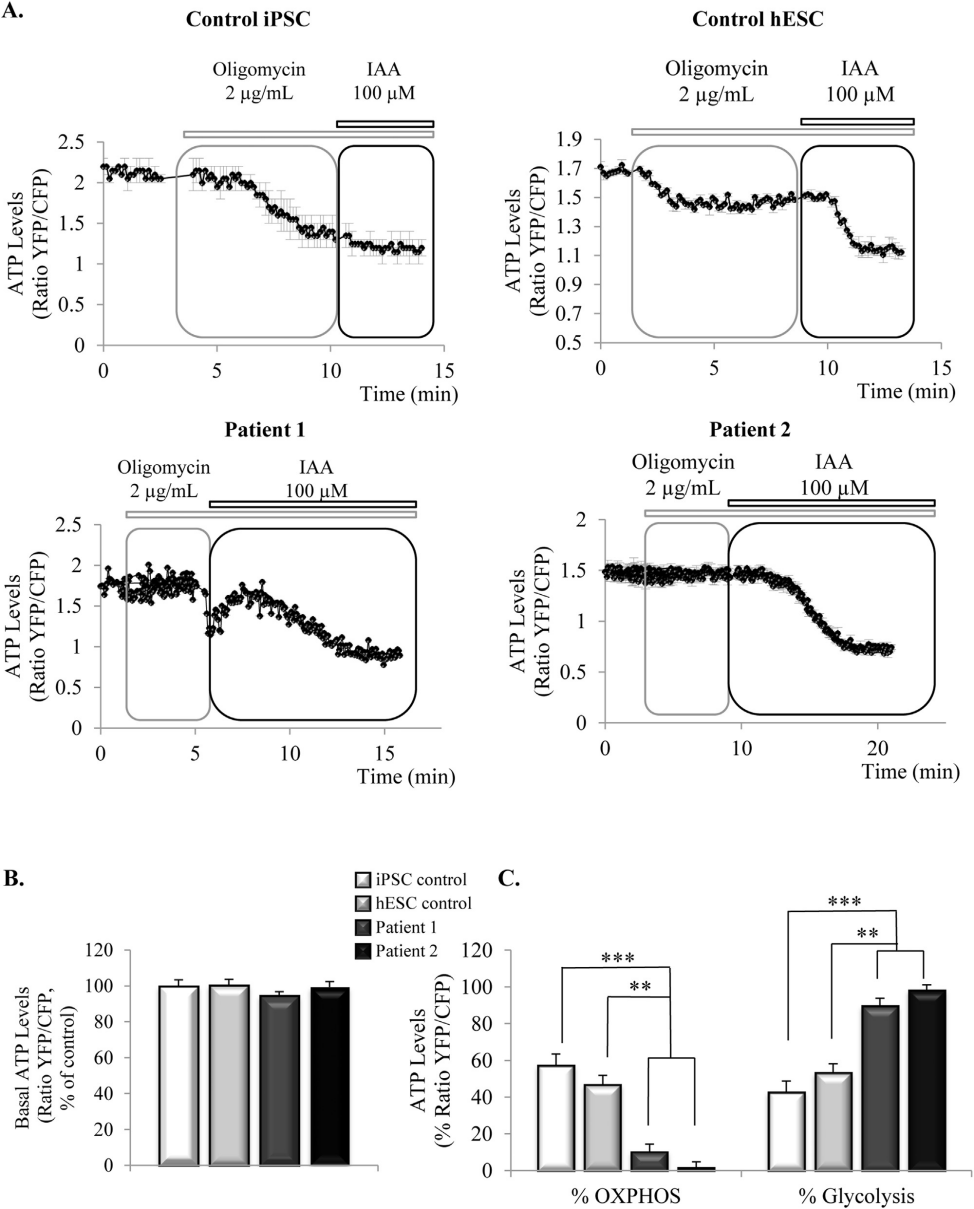
**ATP levels are similar in control and patient cells, but glycolysis is the main source of ATP in the cells of the patients with the*****MAPT*****10+16 mutation**. **3A**. Representative traces showing ATP live measurements. Cells were transfected with AT1.03 probe allowing the visualization of mitochondrial ATP *in vivo.* After measurement of basal levels of ATP (ratio YCF/CFP), oligomycin was added to inhibit complex V. The subsequent reduction in ATP levels can be attributed to ATP generated by oxidative phosphorylation (OXPHOS). Decrease in ATP levels caused by the following addition of iodoacetic acid can be attributed to ATP generated by glycolysis. **3B**. Basal measurements of ATP levels show no difference between controls and patient cells. **3C**. Percentage of ATP produced by OXPHOS or glycolysis, calculated by the percentage of decay caused by each inhibitor. In cells from patients with *MAPT* 10+16 mutation, ATP is mainly produced by glycolysis. (**p<0.01, ***p<0.001).

Mitochondrial respiration is closely coupled to ATP production by oxidative phosphorylation (OXPHOS), so impairment in the respiration may also affect ATP production in neurons with the *MAPT* 10+16 mutation. To evaluate the dynamics and levels of ATP, we transfected the iPSC-derived neurons with the mitochondrial ratiometric ATP indicator designed by [20] (AT1.03) which allows to detect ATP levels in living cells as described in Methods. Despite the lower rates of respiration, neurons from the patients presented similar mitochondrial ATP levels than control neurons (iPSC control, 99.8±3.5%, n=37 cells; hESC control, 100.3±3.4%, n=14 cells; patient 1, 94.5±2.3%, n=40 cells, patient 2, 99.4±3%, n=13 cells) (Fig. 3B).

To have a better understanding of ATP dynamics in this model, we next checked the functioning of the mechanisms of ATP production. There are two main cellular systems implicated in ATP production: glycolysis and oxidative phosphorylation. To study how each of them was contributing to ATP production in the neurons, we sequentially treated them with the inhibitor of complex V oligomycin, and the inhibitor of glycolysis iodoacetic acid (IAA), and analysed the decay in the ATP levels caused by the inhibition of each mechanism, representative traces are shown in 3A. In control cells, inhibition of OXPHOS caused a decay in the ATP levels of 57.3±6.1% and 46.8±5%, with a further decrease of 42.7±6.1 and 53.2±5% after inhibition of glycolysis with IAA (iPSC control cells, n=21 and hESC, n=4, respectively) (Fig. 3C). In contrast, in patient cells, ATP production was mainly dependent on glycolysis as observed by the drop in ATP caused by IAA (patient 1, 89.7±4%; patient 2, 99.4±3%). Complex V inhibition in patient cells produced either a small decrease or a small increase in ATP levels (patient 1, 10.3±4.1%, n=28; patient 2, 1.7±3%, n=8), which can be explained by the inhibition of the hydrolysis of ATP by the F_1_F_o_ATPase working in reverse mode, in agreement with the results shown in Fig. 1D. Results presented in Fig. 3 suggest that the neurons from the patients depend on glycolysis to maintain the ATP levels, rather than on OXPHOS, which is the main source of ATP in iPSC control neurons.

### 3.4 Provision of cells with substrates for TCA cycle increases mitochondrial NADH pool and recuperates the altered bioenergetics features

**Fig. 4:**
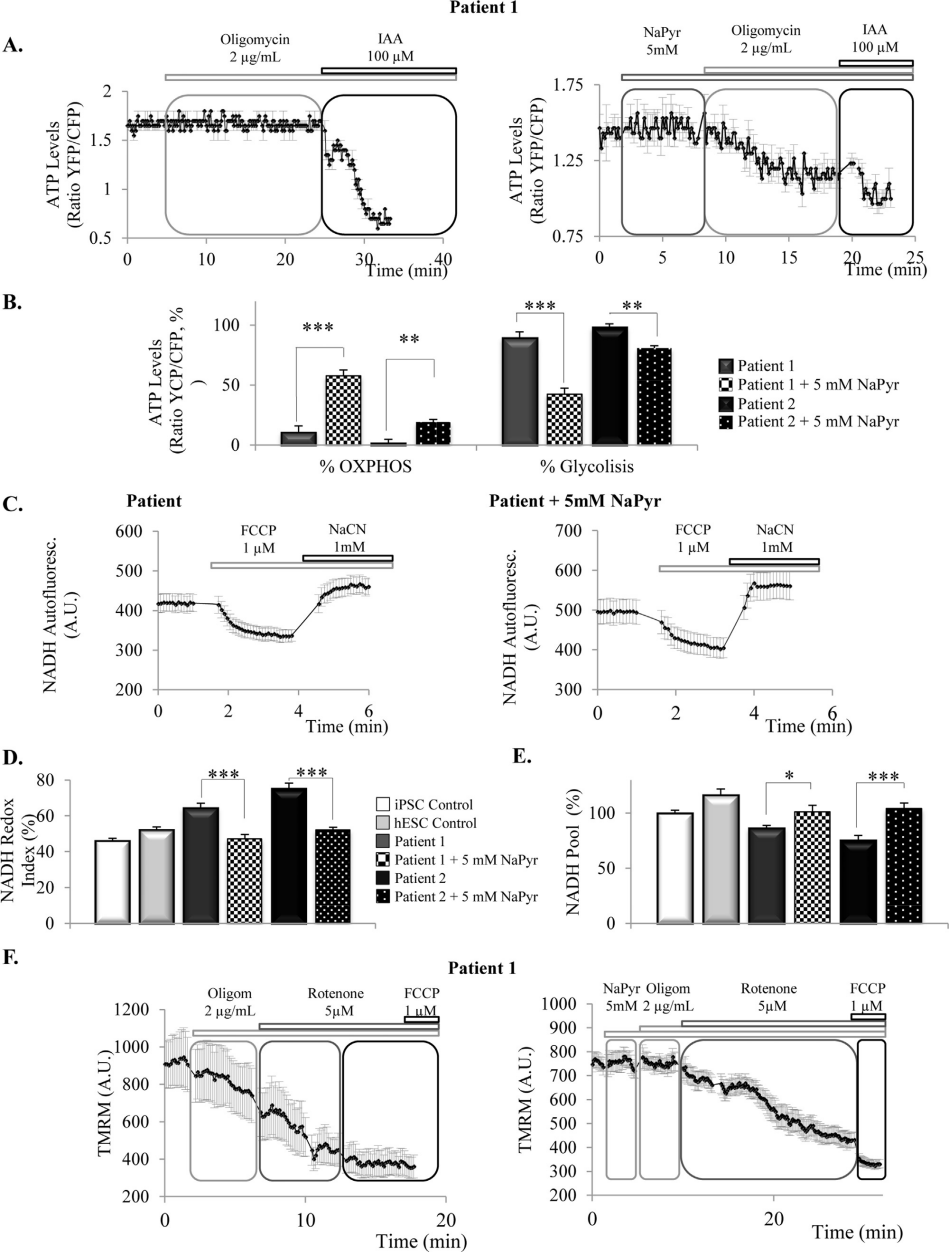
**Provision of patient cells with substrates for TCA cycle increases the mitochondrial NADH pool and recuperates the altered bioenergetics features**. **4A**. Representative traces of ATP live measurements with the ATP AT1.03 construct. **4B**. Quantification of the percentage of ATP produced by oxidative phosphorylation (OXPHOS) (as calculated by the drop in ATP levels caused by oligomycin) and glycolysis (% drop caused by iodoacetic acid) in the cells of the patients in the presence/absence of 5 mM sodium pyruvate (NaPyr) (**p<0.01, ***p<0.001). **4C**. Representative traces of NADH status experiments in patients cells pretreated or not with NaPyr 5 mM. **4D**. Preincubation of the patient cells with NaPyr decreased the NADH Redox index of patient cells to similar levels than controls (***p<0.001). **4E**. Preincubation of the cells of the patients with NaPyr significantly increased the mitochondrial NADH pool (*p<0.05; ***p<0.001). **4F**. Representative traces of mitochondrial membrane potential maintenance in patients’ cells in the absence or presence of NaPyr.

An enhancement of the production of ATP by glycolysis could be a compensatory mechanism for the decreased production of ATP by OXPHOS in iPSC-derived neurons from patients with the mutation in *MAPT,* with the lack of substrates for respiration (Fig. 2C) being the possible underlying cause. To test this possibility, we supplied the cells with pyruvate, to serve as a substrate for the TCA cycle, and checked how ATP was produced under these circumstances; representative traces are shown in Fig. 4A. When patient cells were pre-incubated with 5 mM sodium pyruvate (NaPyr), inhibition of OXPHOS with oligomycin caused a decrease in the ATP levels that was significantly higher than in non pre-treated cells (Fig. 4B: patient 1, pretreated with NaPyr, 57.6±9.5%, n=11 *vs.* non pretreated, 10.7±5.1%, n=8; patient 2, pretreated with NaPyr, 19.2±2.1%, n=4 *vs.* non pretreated 2.3±5.6%, n=4). In parallel, the % of ATP produced by glycolysis was reduced when the patients’ cells were provided with pyruvate (Fig. 4B: patient 1, pretreated with NaPyr, 42.4±9.5% *vs.* non pretreated, 89.3±5.1%; patient 2, pretreated with NaPyr, 80.8±2.1% *vs.* non pretreated, 97.7±5.6%). These results show that in the presence of substrates for the TCA cycle, patient's cells are able to increase their production of ATP by OXPHOS, reducing the ATP produced by glycolysis.

We then confirmed how provision of substrates for the TCA cycle was indeed affecting the respiratory activity, by analysing the NADH redox state in the presence and absence of sodium pyruvate (representative traces are depicted in Fig. 4C). As shown in Fig. 4D, when cells from the patients were incubated with 5 mM of NaPyr, NADH redox index significantly diminished (was more oxidized) in patient 1 from 64.4±2.6% (non treated, n=33) to 47.1±2.4% (treated, n=49) and in patient 2 from 75.4±2.9% (non treated, n=22) to 52.1±1.5% (treated, n=32) (Fig. 4D), indicating that respiration was increased in the presence of substrates for the TCA cycle. Accordingly, the pool of mitochondrial NADH also increased significantly in the presence of sodium pyruvate in the neurons of the patients (Fig. 4E) (patient 1, non treated, 86.4±2.4%, *vs.* treated, 103.3±6%; patient 2, non treated 75.4±4.4% *vs.* treated, 104.1±5%).

Importantly, we also confirmed that the provision of substrates for TCA cycle to the iPSC-derived neurons from FTDP-17 patients completely prevented the oligomycin-induced depolarization of the mitochondrial membrane potential (representative traces shown in Fig. 4F). Drop in ΔΨ_m_ produced by oligomycin decreased from 22±9.1 (patient 1, n=4) and 16.4±2.7% (patient 2, n=15), to 2.2±9.6% (patient 1, n=10) and −7.5±6 (hyperpolarization) in patient 2 (n=15) when cells were pre-treated with NaPyr.

Taken together, these data indicates that in the presence of substrates for the TCA cycle like pyruvate, mitochondrial NADH pool is recovered in iPSC-derived neurons from patients, which turns into higher rates of respiration and ATP produced by oxidative phosphorylation instead of glycolysis. In addition, the electron transport chain is able to maintain the ΔΨm, which is no longer maintained by the complex V working in reverse mode.

### 3.5 ROS production is increased in iPSC-derived neurons carrying the *MAPT* 10+16 mutation

**Fig. 5:**
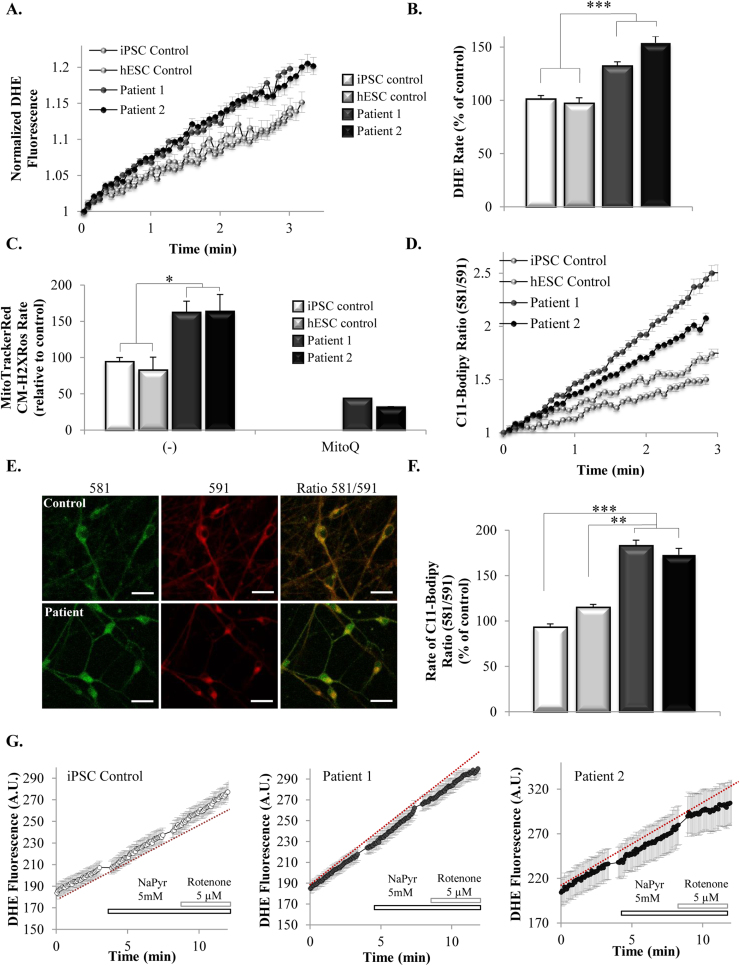
**ROS production and lipid peroxidation are increased in the cells of the patients with the*****MAPT*****10+16 mutation. 5A.** Representative traces showing the rate of cytosolic ROS production as measured by the increase in DHE fluorescence. **5B.** Quantification of the rate of DHE fluorescence, indicating that basal rate of cytosolic ROS production is higher in the cells of the patients compared to the controls (***p<0.001). **5C.** Quantification of the rate of MitoTracker® Red CM-H2Xros, indicating that basal rate of mitochondrial ROS production is increased in the cells of the patients compared to the controls (*p<0.05). **5D.** Representative traces showing the basal rate of lipid peroxidation in the cells using Bodipy 581/591 C11. **5E.** Representative images of C-11 Bodipy fluorescence after 4 min of recording. Oxidation of the dye results in a shift of the fluorescence emission peak from red (~590 nm) to green (~520 nm). Scale bar: 20 µm. **5F.** Quantification of the rate of C-11 Bodipy (581/591), indicating that the basal rate of lipid peroxidation is increased in the cells of the patients (**p<0.01, ***p<0.001). **5G.** Traces showing the effect of substrates (NaPyr) or inhibition of complex I (rotenone) on the rate of ROS production in control and patients neurons.

Despite the partial inhibition of the respiration in patient's cells ATP production is compensated by glycolysis. Considering this, energy deprivation in these cells is unlikely to be a main mechanism of cell death. Reactive oxygen species (ROS) are implicated in the regulation of important physiological functions in the cell [2], [3]. However, overproduction of ROS is considered to play a central role in the pathogenesis of neurodegenerative disorders [11]. Mitochondria are one of the most important intracellular sources of ROS, and both higher mitochondrial membrane potential and alterations in the electron transport chain (as found in iPSC-derived neurons of the patients) can lead to an increase in ROS production [1]. We investigated this feature by measuring the rate of ROS production both in the cytosol and in the mitochondria of the cells. For the first, we used dihydroethidium (DHE), which is a cytosolic ROS indicator predominantly specific for superoxide. Rate of increase in DHE fluorescence upon oxidation of the dye is proportional to the rate of superoxide generation (representative traces in Fig. 5A.) Neurons derived from the patients with 10+16 *MAPT* mutation presented a significantly higher rate of cytosolic ROS production compared to the controls (Fig. 5B, iPSC Control, 101.4±3.2%, n=152; hESC control, 97.5±5%, n=84; patient 1, 132.4±3.7%, n=224; patient 2, 154±6.1%, n=112). The same principle was used to measure the rate of mitochondrial ROS production. To this end, we employed the mitochondrial-targeted dye MitoTracker® Red CM-H_2_XRos, which is a reduced probe that is non-fluorescent until it is oxidized. Fig. 5C shows that the rate of mitochondrial ROS production, calculated as the rate of increase in red fluorescence of the dye, is also significantly increased in the neurons from the patients compared to the controls (iPSC control, 94.4±5.5%, n=6 measurements; hESC control, 82.9±17%, n=4; patient 1, 162.3±15%, n=11; patient 2, 163.9±23%, n=6). To test the specificity of the dye, we also pre-treated cells of the patients with the mitochondria-targeted antioxidant mitoquinone (MitoQ) [22], which induced a dramatic decrease in the rate of mitochondrial ROS production (Fig. 5C).

When imbalanced and not controlled, overproduction of ROS can lead to oxidative stress, which results in damage to macromolecules, membranes and organelles that can be deleterious. Among others, ROS can react with the polyunsaturated fatty acids of lipid membranes, inducing lipid peroxidation, which can also amplify the damage to other macromolecules. We used the ratiometric indicator Bodipy 581/591 C11 to measure the rate of lipid peroxidation. Upon oxidation, the fluorescent properties shift from red to green, allowing the ratiometric measurement of lipid peroxidation (representative traces and images found in Fig. 5D and E). As shown in 5F, the basal rate of lipid peroxidation is significantly increased in the neurons from the patients (iPSC Control, 93.2±3.8%, n=106 cells; hESC Control, 115±3.1%, n=48; patient 1, 183±6.1%, n=211; patient 2, 173±6.9%, n=181), showing that neurons from the patients exhibit higher levels of oxidative stress.

As mentioned before, both the higher mitochondrial membrane potential and alterations in the activity of the respiratory chain can induce an increase in ROS production. To study both mechanisms, we measured the changes in the rate of ROS production using DHE in the presence of a substrate of complex I (5 mM NaPyr) to increase respiration, or in the presence of the complex I inhibitor 5 μM rotenone.

As shown in the representative traces found in Fig. 5F, in the control cells, the application of NaPyr increased the basal rate of ROS production from 96.1±3.7% to 119.6±3.5%, with a further increase to 132.4±5.9% when complex I was inhibited with rotenone (n=123). The cells of the patients, in agreement with Fig. 5B, showed an already increased basal rate of ROS production compared to the controls (patient 1, 167.3±5.4%, n=132; patient 2, 200.6±16.8%, n=29). In those cells, application of NaPyr slightly increased the rate of ROS production (to 153.8±5% in patient 1 and to 205±18% in patient 2) and, in contrast with control cells, rotenone clearly diminished the rate of ROS production to 129±5.4% in patient 1 and to 155.8±19% in patient 2. These results suggest that in the iPSC-derived neurons from the patients with the 10+16 *MAPT* mutation the higher rate of ROS production is sustained by the higher mitochondrial membrane potential rather than by the alterations in the respiratory chain.

### 3.6 Cell death is increased in neurons with the *MAPT* 10+16 mutation and is dependent on ROS production

**Fig. 6:**
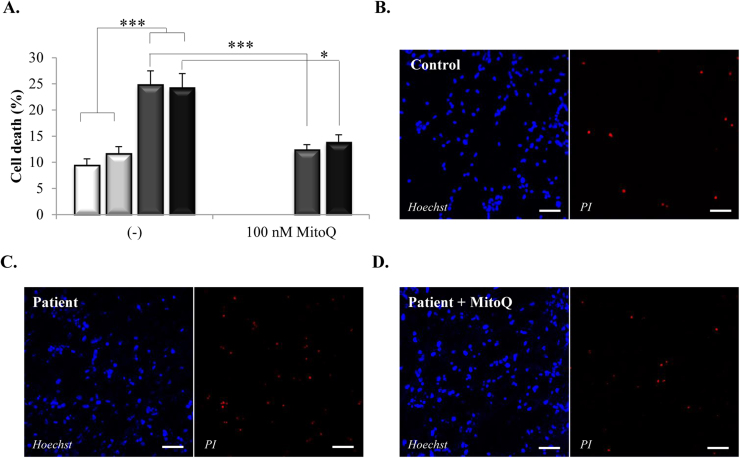
**Cell death is increased in the cells with the*****MAPT*****10+16 mutation and is dependent on ROS production. 6A.** Histogram showing the percentage of cell death in controls and patients in the absence or presence of the inhibitor of mitochondrial ROS, MitoQ, 100 nM.*p<0.05, ***p<0.001**. 6(B–D).** Representative images of control neurons (B), patient neurons (C) and patient neurons treated with MitoQ (D) cell death experiments. Cell toxicity was estimated co-staining the cells with propidium iodide (red fluorescence) which labels dead cells, and Hoechst (blue fluorescence) which labels all cells. Scale bar: 50 µm.

Excessive ROS production can lead to cell death and neurodegeneration, so we next analysed the cell viability and its dependence on the overproduction of ROS in the neurons from the patients. To this end, we co-stained the cells with propidium iodide, which labels dead cells, and Hoechst, which labels all cells, and calculated the percentage of cell toxicity. Representative images are shown in 6B–D. Compared to controls (iPSC control, 9.4±1.2%, n=27; hESC control, 11.6±1.4%, n=19), cells from the patients showed significantly higher basal levels of cell death (patient 1, 24.8±2.7%, n=28; patient 2, 24.2±2.8%, n=2=18), Fig. 6A. Importantly, when we treated the cells of the patients with the mitochondria-targeted antioxidant MitoQ (48 h), which was previously shown to decrease the mitochondrial ROS production in these cells (Fig. 5C), the percentage of dead cells decreased significantly, as shown in Fig. 6A (patient 1+MitoQ, 12.4±1%, n=21; patient 2+MitoQ, 13.8±1.5%, n=10). These results suggest that the excessive ROS production in the cells of the patients, linked to the increased mitochondrial membrane potential, is the underlying cause of the higher cell death found in this model and can be prevented *in vitro* by inhibiting mitochondrial ROS production.

## 4 Discussion

Due to its role in promoting microtubule assembly, recent publications analysing the relationship between tau and mitochondria in iPSC-derived neurons with *MAPT* mutations have focused on the alterations of the axonal transport, vesicle trafficking, mitochondria distribution or neurite growth, as reviewed in [32]. In the present study, we have used iPSC-derived neurons carrying the 10+16 *MAPT* mutation to explore for the first time the mitochondrial bioenergetics and dysfunction caused by tau pathology. We have found that the 10+16 *MAPT* mutation is linked to the hyperpolarization of the mitochondria, which is partially maintained by the complex V working in reverse, leading to an increase in ROS production, oxidative stress and cell death. Moreover, mitochondrial NADH pool is reduced and complex I-driven respiration inhibited, causing a decrease in the ATP production by oxidative phosphorylation that is compensated by an increase in glycolysis.

Mitochondrial dysfunction is a common pathological feature of neurodegenerative disorders and is often characterized by a decrease in the mitochondrial membrane potential [9], which has also been observed in different models of tauopathies [6], [36]. Here, we show that despite their reduced mitochondrial respiration, the iPSC-derived neurons from the patients carrying the 10+16 *MAPT* mutation present an increase in the mitochondrial membrane potential that is partially maintained by the complex V working in reverse mode: pumping protons to the intermembrane space at the expense of ATP hydrolysis. Although surprising, hyperpolarization of the mitochondria has been described before in cells with reduced activity of complex I [1], [10]. Interestingly, a higher ΔΨm has also been observed in isolated mitochondria from the P301L tau mice model [7] and in iPSC-derived motor neurons of the *C9ORF72* model of ALS/FTD [29].

One possible explanation of the increased ΔΨm could involve VDAC (voltage-dependent anion channel). VDAC is present in the mitochondrial outer membrane and regulates the exchange of metabolites between the cytosol and the mitochondria [26]. It has been described that free tubulin binds VDAC [35] and is able to modulate ΔΨm: an increase in free tubulin blocks VDAC and diminishes ΔΨm, while a decrease opens VDAC increasing ΔΨm [30]. 4R isoforms of tau are more effective at assembling microtubules, (which are made of α and β-tubulin dimers) leading probably to a decrease in free tubulin compared to 3R isoforms, and to a potential opening of VDAC that could contribute to the hyperpolarization of the mitochondria.

Impairment of complex I, mitochondrial respiration and oxidative phosphorylation have been previously described for other models of tau pathology, normally leading to decreased ATP levels [34], [36], [6].

Our data indicates that the lower availability of mitochondrial NADH for the complex I results in a diminished mitochondrial respiration and altered ATP production by OXPHOS. Application of pyruvate is able to reverse those defects, providing mitochondrial NADH and increasing respiration. As a result, the cells switch back to oxidative phosphorylation as a source of ATP production and complex V stops working in reverse to maintain the ΔΨm, which is maintained by the electron transport chain in the presence of pyruvate. These results suggest that the functionality of respiratory complexes is maintained in the presence of 10+16 *MAPT* mutation and the reason for the observed lower activity of complex I can be attributed to a diminished availability of substrates. Pyruvate is able to correct the defects shown above, indicating that the lack of substrates for respiration is not caused either by alterations in the TCA cycle. As shown, glucose uptake is not affected by the *MAPT* mutation and glycolysis is efficiently producing ATP as a compensatory mechanism for the decreased OXPHOS, so one possible mechanism that can be altered in the cells of the patients is the activity of the pyruvate dehydrogenase (PDH) complex, which regulates the conversion of pyruvate, the final product of glycolysis, into acetyl-CoA as a first step to enter the TCA cycle. Indeed, lower PDH activity has been shown in an animal model and human brain with AD [38], [45]. Inhibition of the PDH is achieved through its phosphorylation by the different isoforms of the pyruvate dehydrogenase kinase (PDK), which plays an important role in controlling metabolic pyruvate flux [16] and can contribute to different neuropathological conditions [21]. Importantly, high concentrations of pyruvate are able to inhibit PDK, thus activating PDH complex [16], which can explain the recuperation of the bioenergetics alterations when applying pyruvate to the cells of the patients with the *MAPT* mutation.

It has also been described that PDH can be phosphorylated and inactivated by the Alzheimer's disease-related tau protein kinase I (TPKI) [18].

iPSC-derived neurons from the patients of FTDP-17 presented similar levels of ATP despite the reduction in ATP production by OXPHOS, due to an increase in the ATP production by glycolysis. A shift from aerobic respiration to glycolytic metabolism has also been described in several models of Alzheimer's disease as an attempt to maintain ATP production in response to mitochondrial dysfunction [17], [24]. Interestingly, another study found a correlation between spatial distribution of increased aerobic glycolysis and Aβ deposition in brain tissue [43]. Glycolytic enzymes are elevated in AD brains [39] and increased levels of lactate, the final product of aerobic glycolysis have been found in the brain of patients from PSP -another 4R tauopathy- [42] and in the CSF of AD patients [27].

It has also been described that an increase in aerobic glycolysis due to an overproduction of PDK1 (and therefore inhibition of PDH) in nerve cells resulted in a protective effect against β-amyloid toxicity, linked to a reduction in mitochondrial respiration, ΔΨm and ROS, without affecting ATP levels [31], [39]. However, long-term consequences for neuronal survival of the metabolic shift from OXPHOS to glycolysis may be deleterious. Maintenance of ATP levels despite impairment of mitochondrial respiration has been described in P301L *MAPT* mice brain, but resulting in a significant decrease with aging [6]. Our data show that iPSC-derived neurons from the patients are able to maintain ATP levels relying on glycolysis still after 150 days *in vitro*, the most extended time-point used for the experiments. It would be interesting to study if ATP levels are maintained at more extended time-points *in vitro.*

Cell death was however increased in the neurons of the patients with the 10+16 *MAPT* mutation as a result of the increased rate of ROS production in these cells. Higher levels of ROS production and oxidative stress have been reported in different animal and cellular models with *MAPT* mutations [19], [33], [6], generally linked to alterations in the electron transport chain. Recent work done in N279K and V337M models of *MAPT* mutations in iPSC-derived neurons also showed increased oxidative stress in response to respiration inhibition [8]. However, we have found that the increased ROS production in the iPSC-derived neurons with 10+16 *MAPT* mutation was not a result of the impairment of complex I, but instead it was linked to the hyperpolarization of the mitochondria, as shown by the reduction in the rate of ROS production when inhibiting complex I with rotenone. As for mitochondrial membrane potential, differences in the observed phenotype among models with different *MAPT* mutations shouldn’t be surprising giving the wide range of pathologies caused by the different *MAPT* mutations or even the clinical variability within members of a family with the same mutation [12], [14].

Importantly, cell death found in the neurons of the patients was dependent on the increased ROS production, and it could be prevented when treating the patient's cells with the mitochondria-targeted antioxidant MitoQ, which is able to directly scavenge peroxynitrite and superoxide, protecting mitochondria from lipid peroxidation [25]. These results are in agreement with recent work from [29] which also showed that antioxidants could prevent the age-dependent oxidative stress-induced damage in iPSC-derived motor-neurons from ALS/FTD.

In summary, our data provide the first study focused on the bioenergetics and mitochondrial dysfunction caused by tau pathology in iPSC-derived neurons from patients with FTDP-17. Here, we have given new insights to unravel the mechanisms of tau-related neurodegeneration and potential therapeutic strategies for FTDP-17.

## Author's contributions

JDR provided the patient's skin biopsies. JH, SW generated the iPSC lines used in the experiments. NE, JH, SW, AYA conceived the study. NE and AYA designed and performed the experiments and analysed the data. NE wrote the manuscript. All the authors contributed to the final version of the article.

## Conflict of interest

The authors declare no conflict of interest.
